# Combining Procalcitonin and Calcitonin for the Diagnosis of Medullary Thyroid Cancer: A Two‐Step Approach

**DOI:** 10.1111/cen.15287

**Published:** 2025-06-04

**Authors:** Cristina Clausi, Simona Censi, Emma Zanin, Giulia Messina, Ilaria Piva, Daniela Basso, Isabella Merante Boschin, Loris Bertazza, Francesca Torresan, Maurizio Iacobone, Fiammetta Battheu, Susi Barollo, Jacopo Maria Arnone, Caterina Mian

**Affiliations:** ^1^ Department of Medicine (DIMED) University of Padua Padova Italy; ^2^ Endocrinology Unit University Hospital of Padova Padova Italy; ^3^ Laboratory Medicine, Department of Medical and Surgical Sciences University of Padua Padua Italy; ^4^ Endocrine Surgery Unit, Department of Surgical, Oncological and Gastroenterological Sciences (DiSCOG) University of Padua Padua Italy

**Keywords:** calcitonin, diagnosis, markers, medullary thyroid cancer, procalcitonin

## Abstract

**Objective:**

Calcitonin (CT) represents the most important biochemical marker of medullary thyroid cancer (MTC), but has certain limits. Procalcitonin (ProCT) has been recognized as an alternative or additional marker for MTC. The aim of the study is to evaluate prospectively the role of ProCT combined with CT in the identification of MTC.

**Design:**

**Patients and measurements:** 478 patients undergoing thyroidectomy in Padua between January 2023 and June 2024 were enrolled to investigate ProCT levels in comparison with CT for MTC diagnosis. Serum levels of ProCT and CT were dosed preoperatively.

**Results:** At histological diagnosis, 23/478 (4.8%) patients tested positive for MTC. CT with a cut‐off > 10 pg/mL performed as follows: sensitivity 0.91, specificity 0.98, positive predictive value (PPV) 0.7, negative predictive value (NPV) 0.99. CT with a cut‐off > 10 pg/mL performed better than ProCT both using the cut‐off of 0.04 ng/mL (sensitivity 0.87; specificity 0.96; PPV 0.56; NPV 0.99) and the cut‐off of 0.07 ng/mL (sensitivity 0.78; specificity 0.98; PPV 0.72; NPV 0.99). Within the sample of patients with a CT value between 10 and 100 pg/mL, 17/21 (80.9%) patients would have been correctly identified as MTC or non‐MTC based on a positive or negative ProCT using the 0.04 ng/mL cut‐off.

**Conclusions:** CT is more sensitive than ProCT as a diagnostic marker for MTC. However, a two‐step approach using ProCT as a supplementary marker can help to refine the diagnosis avoiding overtreatment, particularly when CT serum levels lie between 10 and 100 pg/mL.

## 1 Introduction

MTC originates from parafollicular C‐cells in the thyroid, accounting for 2% of all thyroid malignancies and 0.4%–1.4% of all thyroid nodules [1]. MTC occurs sporadically in 75%–80% of cases and in an hereditary form in 20%–25% of cases, linked to *RET* (REarranged during Transfection) gene and multiple endocrine neoplasia 2 (MEN2) syndrome. Treatment of MTC is total thyroidectomy with central lymph node dissection, with more extensive surgery required in the presence of extrathyroidal spread [2]. Survival is strongly correlated with stage of disease at the time of diagnosis. The 10‐year survival rate ranges from 100% in stage I to 20% in stage IV, emphasizing the need for early diagnosis [3]. Most patients with MTC present with a thyroid nodule [4], but MTC lacks specific ultrasound (US) features. Approximately 50% of MTC may not be classified as high‐risk on US [5], and also fine needle aspiration (FNA) sensitivity in patients with MTC nodules is far from accurate (56% to 69%) [6, 7]. FNA‐CT has demonstrated remarkable sensitivity and apparently better diagnostic performance, but lacks standardized threshold and risks false positives in C‐cell hyperplasia [8]. Timely and early recognition significantly impacts MTC outcomes, making biochemical diagnosis crucial. CT is highly sensitive in the diagnosis and follow‐up of MTC, but its specificity is low due to influences from various physiological and pathological conditions, such as age, male sex, smoking, drugs, other neuroendocrine tumors, pancreatic cancer, breast cancer, small cell lung cancer, systemic mastocytosis, hyperparathyroidism and chronic renal failure [9, 10, 11, 12]. Conflicting data are available on autoimmune thyroiditis and thyroid tumors of follicular origin and chronic hypergastrinemia [13]. CT has also many analytical problems: a short and concentration‐dependent half‐life at room temperature (15–40 min), susceptibility to laboratory interferences [14, 15] and, due to the large number of kits available in the market, CT results cannot be compared between different laboratories or assays [15]. No consensus exists on CT cut‐off values for MTC diagnosis and follow‐up [2]. While CT levels above 100 pg/mL have a 100% PPV for MTC, levels between 10 and 100 pg/mL have only a 25% PPV [16], thus requiring further evaluations, including calcium gluconate stimulation test or CT measurement from thyroid nodule eluate [17]. Worth of note, however, that the sensitivity and specificity of CT depend also on the resection rate. The higher the resection rate, the higher the MTC rate, the higher CT assay's analytic sensitivity [18].

However, data in the literature on baseline and stimulated CT thresholds are not uniform [19]. Due to these limitations, studies on new, more specific markers are needed. The carcinoembryonic antigen (CEA) has limited diagnostic utility: it is not a specific marker [20] and it is elevated at diagnosis only in 60%–70% of MTC patients [10, 11, 12, 21]. However, CEA is considered particularly useful in the follow‐up in cases of MTC dedifferentiation, when a rising CEA level is observed, accompanied by a stable or decreasing CT trend [20, 21]. ProCT, the prohormone of CT, has been proposed as an alternative tumor marker in the diagnosis and follow‐up of MTC [14, 22] ProCT overcomes many CT limitations, with a stable, concentration‐independent half‐life of 20–24 h at room temperature [23] and less variability across assays due to standardized licensing [24, 25]. ProCT levels are not influenced by gender, physiological and pathological conditions or drugs that increase CT levels [26] and it is useful in cases of spurious hypercalcitoninemia caused by heterophilic antibody interference [27, 28]. Furthermore, rare cases of CT‐negative and ProCT‐positive MTC have been documented [29]. In the absence of signs of inflammation, ProCT, like CT, is produced only by parafollicular cells and neuroendocrine cells [30]. ProCT correlates with CT in pre‐surgical settings, reflecting tumor burden and prognosis [22, 31], and in postsurgical settings, with increased ProCT in most recurrent MTCs [23, 32]. Indeed, no universally recognized ProCT cut‐offs exist beyond those developed for sepsis, which may not be optimal for neoplasia [33]. A retrospective study by Censi et al proposed a baseline ProCT cut‐off of 0.07 ng/mL with sensitivity, specificity, PPV and NPV of 85.7%, 98.9%, 98.2% and 90.6%, respectively [22]. However, thresholds balancing specificity and sensitivity remain undefined. Furthermore, ProCT appears not to be sensitive in identifying medullary microcarcinomas [33].

Thus, the aim of the present study is to evaluate prospectively the role of procalcitonin combined with calcitonin, in the diagnosis of medullary thyroid cancer.

## 2 Materials and Methods

### 2.1 Patients

We conducted a prospective study on patients undergoing thyroidectomy between January 2023 and June 2024 for nodular thyroid disease. The project was approved by the Ethics Committee for Clinical Trials (CESC) of the Padua Hospital and conducted in accordance with the guidelines of the Declaration of Helsinki and the Good Clinical Practice standards of the European Union. Recruitment took place at the Endocrine Surgery Unit of Padua University Hospital (approval code: 486n/AO/24). Inclusion criteria were: age ≥ 18 years; thyroid surgery performed for nodular disease, both benign or malignant; ability to express informed consent. Exclusion criteria were: inability to express informed consent.

Clinical, laboratory and pathological data were collected by consulting the online records. TNM data were evaluated according to the eighth edition of the American Joint Committee on Cancer staging (2017) [34].

### 2.2 Laboratory Assays

CT was assayed using a LIAISON CT II‐GEN two‐site chemiluminescent immunoassay (CLIA) (DiaSorin Inc., Stillwater, MN, USA), with an analytical sensitivity of 2 pg/mL.

ProCT was assayed using a LIAISON BRAHMS PCT II GEN two‐site, two‐step CLIA (DiaSorin, Saluggia, Italy) with an analytical sensitivity of 0.04 ng/mL.

We evaluated basal CT positivity considering a reference upper limit of 10 pg/mL adopted at our institution and considering the sex‐specific cut‐off, based on the study by Fugazzola et al, of > 30 pg/mL and > 34 pg/mL in females and males, respectively [19].

The positivity of ProCT levels were considered both using a cut‐off > 0.04 ng/mL (if detectable) and a cut‐off > 0.07 ng/mL, identified previously by our group [22].

### 2.3 Statistical Analysis

Data were processed using statistical software (R package version 2.7‐2). First, the normality of the distribution was analyzed using the Shapiro‐Wilk test and it was observed that CT and ProCT values were distributed in a non‐normal way. For this reason, CT and ProCT values were expressed as median values (with interquartile range, IQR). The chi‐square test was used to analyze the associations between the diagnosis of MTC and non‐MTC histology with respect to the various CT and ProCT cut‐offs. The Receiver Operating Characteristic (ROC) analysis was used to search for the best cut‐off for the diagnosis of MTC. A value of *p* < 0.05 was considered statistically significant.

## 3 Results

### 3.1 Baseline Characteristics

The final series consisted of 478 subjects: 384 (80.3%) females and 94 (19.7%) males. The median age was 56.7 years (age range 15.1–89.9 years, IQR: 44.6–65.7 years). The definitive histological diagnosis saw the presence of 36/478 (7.5%) adenomas, 2/478 (0.4%) metastases from renal carcinomas, 1/478 (0.2%) lymphoma, 210/478 (43.9%) papillary carcinomas, 27/478 (5.6%) follicular carcinomas, 2/478 (0.4%) intra‐thyroid parathyroid carcinomas, 2/478 (0.4%) poorly differentiated carcinomas, 23/478 (4.8%) MTC, 169/478 (35.5%) goiters and 6/478 (1.3%) thyroiditis. Restricted to patients with MTC, 14/23 (60.9%) were females and 9/23 (39.1%) were males, with a median age of 59.9 years (age range 40.4–79.4 years, IQR: 53.7–64.6 years). MTC stage was available for all 23 patients, accordingly: 12/23 patients at stage 1 (52.2%), 2/23 patients at stage 2 (8.7%), 4/23 patients at stage 3 (17.4%) and 5/23 patients at stage 4 (21.7%). Among MTC patients, 21/23 (91.3%) had a CT > 10 pg/mL and 14/23 (60.9%) were positive for the sex‐specific cut‐offs. Regarding ProCT, 20/23 (87.0%) patients were above the cut‐off of 0.04 ng/mL and 18/23 (78.3%) patients were above the cut‐off of 0.07 ng/mL. Two patients, despite were carriers of MTC, had CT < 10 pg/mL and ProCT < 0.04 ng/mL.

Table 1 shows the clinical and histological characteristics of patients with a final diagnosis of MTC.

**Table 1:** Baseline characteristics of patients diagnosed with MTC.

Sex	Age	FNA	T	N	M	Stage	Surgical operation	ProCT (ng/mL)	CT (pg/mL)	Size (mm)
F	66	TIR5	1b	0	0	1	TT + CLND	2.22	452	18
M	77	TIR1	1a	0	0	1	TT + CLND	0.05	38.9	2
F	58	TIR5	1b	0	0	1	TT + CLND	3.29	255	17
F	45	TIR3B	1b (m)	1a	0	3	TT + CLND	2.38	67.3	15
F	73	TIR3B	1b (m)	1a	0	3	TT + CLND	3.1	374	13
M	50	TIR5	1a	1a	0	3	TT + CLND	3.02	152	9
F	64	TIR4	1b	1a	0	3	TT + CLND	4.28	1790	17
M	53		1a	1b	0	4 A	TT + CLND + left lateral cervical lymphectomy	8.07	187	8
M	62	TIR4	2	1b	0	4 A	TT + CLND + left lateral cervical lymphectomy	6.16	1440	21
M	41	TIR5	1b (m)	1b	0	4 A	TT + CLND + left lateral cervical lymphectomy	9.37	1110	11
M	71	TIR5	3a (m)	1b	1	4 C	ET + CLND + left laterocervical lymphectomy with jugular vein removal/excision	484	11200	65
M	54	TIR4	1a (m)	x	0	1	TT + CLND	0.44	45.2	8
F	68	TIR5	1b	0	0	4	TT + CLND + bilateral lateral cervical lymphectomy	1.17	32.5	11
F	80	TIR2	1a	0	0	1	TT + CLND	0.29	99.5	6
F	52	TIR3B	1a	x	0	1	TT + CLND	0.04	1	2.5
F	61	TIR2	1a (m)	0	0	1	TT + CLND	0.04	17.4	9
F	46	TIR4	1a	0	0	1	TT + CLND	0.23	27.1	3
M	52	TIR1	1a	0	0	1	TT + CLND	0.1	30.8	7
F	62	TIR4	1a	0	0	1	TT + CLND	16	28.7	5
M	39		1a (m)	0	0	1	TT + CLND	0.24	16.5	3
F	57	TIR3A	1a	0	0	2	TT + CLND	0.04	1.2	2
F	61	TIR5	2	0	0	2	TT + CLND	6.37	16.7	21
F	53	TIR3A	1a	x	0	1	TT + CLND	0.07	22.6	8

Abbreviations: CLND, central lymph node dissection; CT, calcitonin; FNA, fine needle aspiration; HT, hemithyroidectomy, ProCT, procalcitonin; TT, total thyroidectomy.

### 3.2 Calcitonin Performances in Diagnosing of MTC

The median value of CT in all patients was 1.0 pg/mL (IQR: 1.0–3.0 pg/mL), with a maximum value of 11200 pg/mL. CT was > 10.0 pg/mL in 30/478 patients (6.3%). The median value of CT in MTC was 45.2 pg/mL (IQR: 24.8–314.5 pg/mL).

**Figure 1:**
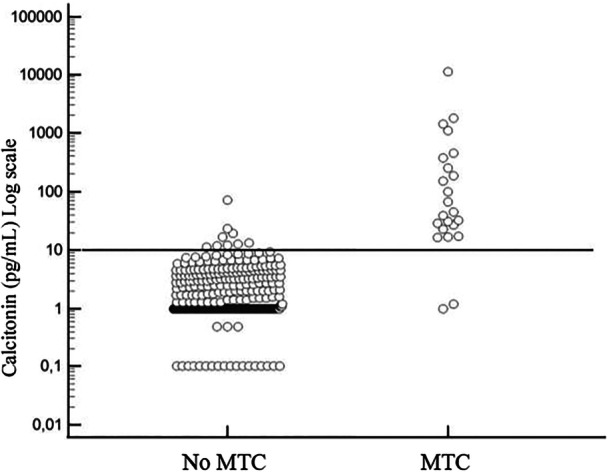
Scatter plot of CT serum concentrations in all patients. The horizontal dashed line represents the CT cut‐off value of 10 pg/mL. MTC: medullary thyroid carcinoma.

Among patients with MTC, 2/23 (8.7%) had CT < 10 pg/mL, while 9/455 (2.0%) patients without MTC had CT > 10 pg/mL (Figure 1).

CT > 10 pg/mL had a sensitivity of 0.91, a specificity of 0.98, a PPV of 0.70, an NPV of 0.99 and an accuracy of 0.98 for the diagnosis of MTC.

**Figure 2:**
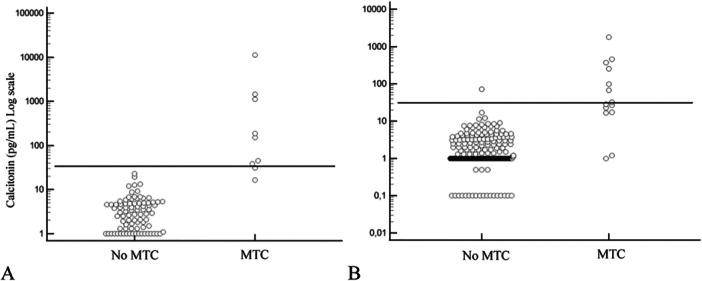
Scatter plot of CT serum concentrations in all patients. The horizontal dashed lines represent sex‐specific cut‐offs values of CT: (A) Male, (B) Female. MTC: medullary thyroid carcinoma.

Using sex‐specific cut‐offs [19], CT was positive in 15/478 (3.1%). In the sample of patients with MTC, 9/23 (39.1%) patients had a negative CT. Only one female patient out of 455, despite not having an MTC, was positive considering sex‐specific CT cut‐offs (Figure 2B). The sex‐specific cut‐offs of CT therefore showed the following performance: sensitivity 0.61; specificity 0.99; PPV 0.93, NPV 0.98 and accuracy 0.98.

### 3.3 Procalcitonin Performances in Diagnosis of MTC

**Figure 3:**
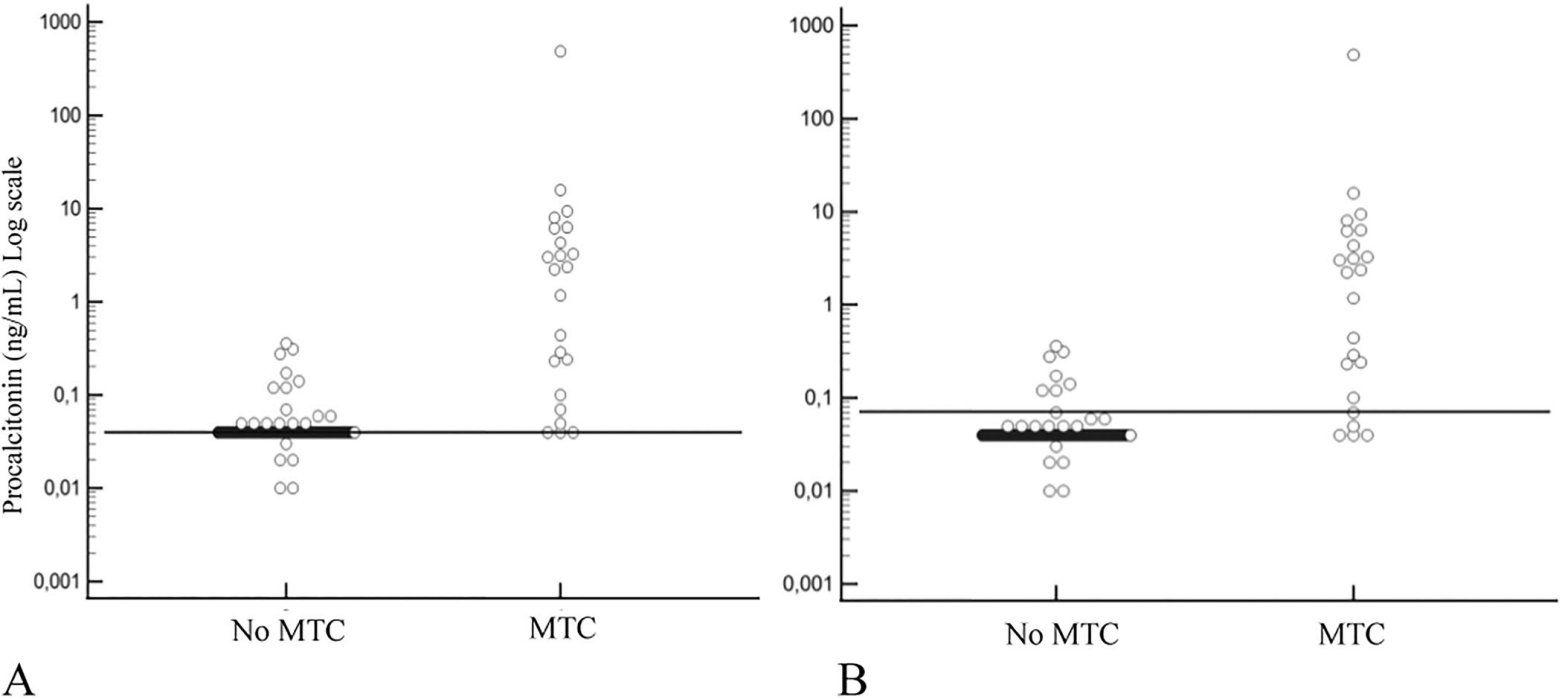
Scatter plot of ProCT serum concentrations in all patients. The horizontal dashed lines represent ProCT cut‐off values. (A) ProCT cut‐off of 0.04 ng/mL, (B) ProCT cut‐off value of 0.07 ng/mL. MTC: medullary thyroid carcinoma.

The median value of ProCT in all patients was 0.04 ng/mL (IQR 0.04–0.04 ng/mL), with a maximum value of 484.0 ng/mL. In patients with MTC, median ProCT value was 2.22 ng/mL (IQR: 0.16–5.22 ng/mL). Using a ProCT cut‐off of 0.04 ng/mL, 36/478 patients (7.5%) tested positive for ProCT. Among patients with MTC, 20/23 (87.0%) tested positive for ProCT, while 439/455 (96.5%) patients without MTC tested negative for ProCT. Additionally, 16/455 (3.5%) had ProCT above the cut‐off but were free of MTC (Figure 3A). The following accuracy parameters were obtained: sensitivity 0.87, specificity 0.96, PPV 0.56, NPV 0.99, accuracy 0.96. Using ProCT cut‐off identified in our group's previous work [22] for baseline ProCT (ProCT > 0.07 ng/mL), 25/478 (5.2%) patients tested positive for ProCT. In this case, 18/23 (78.3%) patients with MTC tested positive for ProCT, while 448/455 (98.5%) patients without MTC tested negative. Furthermore, 7/455 (1.5%) patients had ProCT above the cut‐off even though they were free of MTC (Figure 3B). The following accuracy parameters were obtained: sensitivity 0.78, specificity 0.98, PPV 0.72, NPV 0.99, accuracy 0.97.

**Figure 4:**
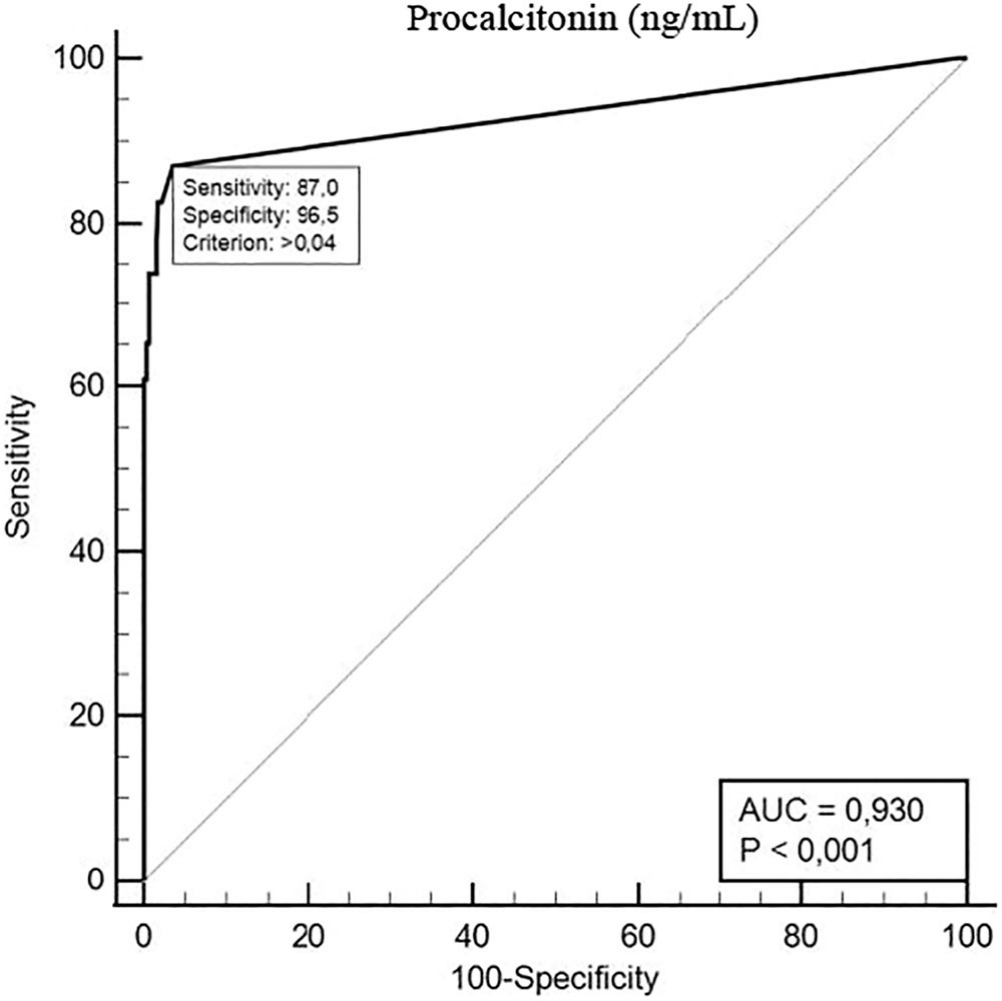
ROC curve analysis to identify the cut‐off levels for ProCT with the highest accuracy to differentiate between non‐MTC and MTC.

Following ROC curve analysis, the best ProCT cut‐off to differentiate MTC from non‐MTC was > 0.04 ng/mL (sensitivity 86.9%, specificity 96.5%, PPV 65.5%, NPV 99.3% AUC 0.93 *p* < 0.0001) (Figure 4).

### 3.4 The Combined Approach in the Diagnosis of MTC

Results of combined approach using CT plus ProCT in the diagnosis of MTC, according to the different cut‐offs of positive basal values, were reported in Table 2.

**Table 2:** Diagnostic performance of combined approach using CT plus ProCT in the diagnosis of MTC, according to the different cut‐off of positive basal values.

		MTC (23)	NON MTC (455)	
**CT** > **10 pg/mL + ProCT** > **0.04 ng/mL**	Positive	20/23	3/455	**Sensitivity** 0.87
	Negative	2/23	433/455	**Specificity** 0.95
	Discordant (CT + /ProCT‐)	1/23	6/455	**PPV** 0.48
	Discordant (CT‐/ProCT + )	0/23	13/455	**NPV** 0.99
				**Accuracy** 0.95
**CT > sex‐specific cut‐offs + ProCT** > **0.04 ng/mL**	Positive	14/23	1/455	**Sensitivity** 0.61
	Negative	3/23	439/455	**Specificity** 0.96
	Discordant (CT + /ProCT‐)	0/23	0/455	**PPV** 0.47
	Discordant (CT‐/ProCT + )	6/23	15/455	**NPV** 0.98
				**Accuracy** 0.95
**CT** > **10 pg/mL + ProCT** > **0.07 ng/mL**	Positive	18/23	2/455	**Sensitivity** 0.78
	Negative	2/23	441/455	**Specificity** 0.97
	Discordant (CT + /ProCT‐)	3/23	7/455	**PPV** 0.56
	Discordant (CT‐/ProCT + )	0/23	5/455	**NPV** 0.99
				**Accuracy** 0.96
**CT > sex‐specific cut‐offs + ProCT** > **0.07 ng/mL**	Positive	13/23	1/455	**Sensitivity** 0.57
	Negative	4/23	448/455	**Specificity** 0.98
	Discordant (CT + /ProCT‐)	1/23	0/455	**PPV** 0.65
	Discordant (CT‐/ProCT + )	5/23	6/455	**NPV** 0.98
				**Accuracy** 0.96

Abbreviations: CT, calcitonin; ProCT, procalcitonin; PPV, positive predictive value; MTC, medullary thyroid cancer; NPV, negative predictive value.

As observed from the data presented, when ProCT is used as an additional marker for the diagnosis of MTC, the diagnostic accuracy remains the same regardless of whether a cut‐off value of CT > 10 pg/mL is used, or if sex‐specific cut‐offs are applied. This means that the effectiveness of the ProCT in correctly identifying MTC does not significantly change between using an universal threshold of CT > 10 pg/mL or adjusting the threshold based on sex‐specific values.

### 3.5 ProCT in Patients With Moderately Elevated CT Levels (10–100 pg/mL and 20–100 pg/mL)

In our sample, 21 patients had a CT between 10 and 100 pg/mL, of which 12/21 (57.1%) were carriers of MTC and 9/21 (42.9%) had no MTC. In these 12 patients with MTC, there were 8 females and 4 males. Using a ProCT cut‐off of 0.04 ng/mL, 11/12 (91.7%) patients would have been correctly identified by ProCT. Using a ProcCT cut‐off of 0.07 ng/mL, 9/12 (75.0%) patients would have been correctly identified by ProCT. In patients without MTC, 6/9 (66.7%) had a ProCT under 0.04 pg/mL and 7/9 (77.8%) had a ProCT under 0.07 ng/mL. Consequently, 17/21 (80.9%) with CT levels > 10 pg/mL but < 100 pg/mL would have been correctly identified as MTC or non‐MTC based on a positive or negative ProCT compared to the 0.04 ng/mL cut‐off, while using a ProCT value of 0.07 ng/mL, 16/21 (76.2%) patients would have been correctly identified as MTC or non‐MTC.

**Figure 5:**
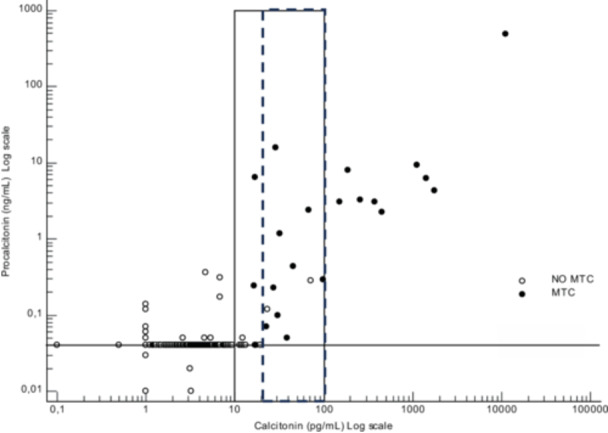
Correlation between serum CT and ProCT in MTC patients and non‐MTC patients. The horizontal dashed line represents the ProCT cut‐off value of 0.04 ng/mL. The solid‐line rectangle represents the CT concentration range of 10 to 100 pg/mL, while the dashed‐line rectangle indicates the CT concentration range of 20 to 100 pg/mL. Samples from the MTC group are depicted by solid symbols. The open symbols depict the samples from the non‐MTC groups. MTC, medullary thyroid carcinoma.

A meta‐analysis of Piticchio et al [18] has demonstrated that the MTC rate found among patients with CT between 10 and 20 pg/mL was significantly lower than that observed among patients with CT between 20 and 100 pg/mL. Thus, we assess the role of ProCT as an ancillary marker also among patients with indeterminate CT values among 20 and 100 pg/mL. In this prospective series of 478 thyroidectomies, 2.3% (11/478) had a CT values > 20 pg/mL but < 100 pg/mL. Among these, 9/11 (82%) had MTC and 2/11 (18%) were free of MTC. Using a ProCT cut‐off of 0.04 ng/mL, 9/9 (100%) patients would have been correctly identified by ProCT. Using a ProCT cut‐off of 0.07 ng/mL, 7/9 (77.8%) patients would have been correctly identified by ProCT. In patients without MTC, 0/2 (0%) had a ProCT under 0.04 ng/mL and under 0.07 ng/mL. Consequently, 9/11 (81.8%) with CT levels > 20 pg/mL but < 100 pg/mL would have been correctly identified as MTC or non‐MTC based on a positive or negative ProCT using the 0.04 ng/mL cut‐off, while using a ProCT value of 0.07 ng/mL, 7/11 (63.6%) patients would have been correctly identified as MTC or non‐MTC (Figure 5).

## 4 Discussion

CT has been used for years in the diagnosis and follow up of MTC. However, conclusive evidence regarding its application as a screening marker in the work‐up of thyroid nodes are lacking [2]. While CT can detect almost all cases of MTC using a cut‐off > 10 pg/mL [35], the low frequency of MTC (0.4%–1.4% of all thyroid nodules) [1], and the low specificity for a whole series of secondary causes, whether pathological or physiological [15], could lead to unnecessary additional tests as well as unnecessary thyroidectomies. To minimize overtreatment, sex‐specific cut‐offs for CT have been suggested [19]. However, this approach inevitably results in decreased sensitivity; furthermore, no shared sex‐specific cut‐offs are provided according to different studies in Literature [19, 36, 37, 38, 39, 40, 41]. Indeed, CT values ranging between 10 and 100 pg/mL remain a recurrent diagnostic challenge in the routine clinical practice, often resulting in false positives issues, which can cause potential overtreatment and unnecessary patient's follow‐up. In these cases, a calcium gluconate stimulation test can improve CT specificity and confirm the suspicion of MTC. However, the test is only accessible at tertiary level centers with personnel experienced in its execution and, once again, no reliable and universally accepted cut‐offs are available [19, 36, 37, 38, 39, 40, 41]. These limitations, in conjunction with the numerous analytical drawbacks of CT, have led to the pursuit of better markers. Previous studies have shown the non‐inferiority of ProCT compared to CT for diagnosing MTC [14, 22, 23]. Moreover, ProCT has many characteristics that overcome CT pitfalls, making it a promising alternative marker in routine clinical practice [33]. So, the aim of our study was to evaluate the performance of ProCT alone and in combination with CT in the diagnosis of MTC in a real‐word prospective series, carried‐out in a medical research institute.

The results showed that ProCT alone is not a better diagnostic marker for MTC than CT, also when its specificity and PPV are considered. In our study, we show that ProCT alone, when compared with CT > 10 pg/mL, appears to be less sensitive both when considering the cut‐off of 0.04 ng/mL (sensitivity CT > 10 pg/mL 0.91 Vs ProCT > 0.04 ng/mL 0.87) and when considering the cut‐off of 0.07 ng/mL (sensitivity CT > 10 pg/mL 0.91 Vs ProCT > 0.07 ng/mL 0.78). Considering only the ProCT with the cut‐off of 0.04 ng/mL, our study highlighted that three patients escape the diagnosis of MTC: one of these has a ProCT below the cut‐off but a CT > 10 pg/mL, while two patients exhibited negative results on both markers. Using ProCT with a cut‐off of 0.07 ng/mL, five MTC cases were missed. However, by using CT with a cut‐off value of 10 pg/mL, it was possible to identify MTC in three of these five patients, while the remaining two were negative for both markers. It is important to point out that in these two patients the MTC were ≤ 10 mm in size, in line with the findings from the 2021 study by Censi et al. which states a lower sensitivity of ProCT for microcarcinomas (65.2% if ≤ 10 mm) [22]. This further confirms that ProCT should not be used as a standalone diagnostic marker. Given the importance of a timely diagnosis of MTC to improve its outcome, the role of ProCT should remain ancillary to CT. Interestingly and at variance with the retrospective research conducted by Censi et al [22] and the prospective study of Giovanella et al, [42] our prospective and real‐life series shows that the specificity of ProCT is not as strong; in fact, our findings indicate that CT is more specific than ProCT. This may be attributed to the higher number of patients in our study who underwent thyroidectomy by comparison with other two previous studies. Thus, in a real‐life prospective setting, the specificity of ProCT appears to be lower than formerly expected.

When ProCT is utilized as an additional marker, applying a cut‐off of CT > 10 pg/mL or using sex‐specific cut‐offs values of CT, yields similar results in terms of accuracy. Consequently, incorporating ProCT as an additional marker alongside CT would remove the need for sex‐specific CT cut‐offs, which differ widely between studies and lack universal applicability.

Remarkably, with a two‐step approach, our data indicate that ProCT may play a supportive role as an additional marker to improve the diagnostic performance of CT, particularly in the gray zone, representing a true clinical dilemma in clinical practice.

In this prospective series of 478 thyroidectomies, 81% of patients with CT levels > 10 pg/mL but < 100 pg/mL would have been correctly identified as MTC or non‐MTC based on a positive or negative ProCT compared to the 0.04 ng/mL cut‐off, while using a ProCT value of 0.07 ng/mL, 76% of patients would have been correctly identified as MTC or non‐MTC. We the accessed the role of ProCT as an ancillary marker also among patients with indeterminate CT values 20–100 pg/mL, in which a higher rate of MTC is expected, according to Piticchio et al. [18] findings. In this case, 81.8% patients would have been correctly identified as MTC or non‐MTC based on a positive or negative ProCT using the 0.04 ng/mL cut‐off. Consequently, integrating ProCT with CT in the diagnosis of MTC, using the two‐step approach, can help to obtain an early diagnosis avoiding overtreatment. However, it must be emphasized that ProCT cannot be used as a diagnostic marker alone, but only as an additional marker to CT and that, the rate of MTC certainly influences cut‐offs performances.

Our study has several limitations. First, it considered a consecutive and prospective series of thyroidectomies, coming from a medical research institute, a reference point for surgical intervention in case of thyroid disorders, particularly MTC. This leads to a high prevalence of MTC (4.80%), compared to an expected prevalence of around 0.3% [33]. This has certainly altered the test performances in terms of PPV and NPV. Furthermore, any septic states, especially of bacterial origin, which could alter the ProCT values, were not considered, nor the possible use of interfering drugs such as PPIs, glucocorticoids, and beta‐blockers which can instead alter the CT values. Moreover, the use and the performance of a calcium gluconate stimulation test in the setting of moderately elevated CT levels have not been considered.

## 5 Conclusion

In conclusion, according to the results of our study, CT would still appear to be a more sensitive and specific marker than ProCT for MTC diagnosis. ProCT should not be used as an independent diagnostic marker. Considering the critical importance of early and accurate diagnosis of medullary thyroid carcinoma MTC to improve patient outcomes, the role of ProCT should remain strictly ancillary to CT. However, a two‐step approach using ProCT as a supplementary marker could enhance the diagnostic performance in case of a moderately elevated CT finding. Patients with thyroid nodules exhibiting mildly elevated CT levels but have undetectable ProCT have a low probability of carrying an MTC and their follow‐up can be less stringent, thus potentially reducing health care costs.

## Conflicts of Interest

The authors declare no conflicts of interest.

## Data Availability

The data supporting the findings of this study are available on request from the corresponding author, Simona Censi.
